# The Effect of Laser Power on the Properties of M_3_B_2_-Type Boride-Based Cermet Coatings Prepared by Laser Cladding Synthesis

**DOI:** 10.3390/ma13081867

**Published:** 2020-04-16

**Authors:** Zhaowei Hu, Wenge Li, Yuantao Zhao

**Affiliations:** Merchant Marine College, Shanghai Maritime University, Shanghai 201306, China; huzw0731@sina.com

**Keywords:** boride-based cermet, laser cladding synthesis, laser power, microstructure, hardness, corrosion resistance, wear resistance

## Abstract

Boride-based cermet can serve as a good protective coating for low-corrosion and wear-resistant materials, such as carbon steels, due to their mechanical and chemical properties. In this study, M_3_B_2_ (M: Mo, Ni, Fe, and Cr) boride-based cermet coatings were fabricated on Q235 steel with mixed powders of Mo, B, Ni60, and Cr by laser cladding synthesis, and the effects of laser power on the properties of the cermet layer were investigated. Three laser powers (2200, 2500, and 2800 W) were used at the same scanning speed. The X-ray diffraction (XRD), scanning electron microscopy (SEM), and energy-dispersive X-ray spectroscopy (EDS) analysis confirmed that all the coatings were composed of M_3_B_2_-type borides and {Fe, Ni} alloys. The micro-hardness, corrosion, and frictional experiments showed that the cermet coatings enhanced the corresponding performances of the Q235 steels at the three laser powers. However, the micro-hardness of the coatings decreased as the power increased, and the maximum micro-hardness value was 1166.3 HV (Vickers Hardness). The results of the corrosion and frictional experiments showed that the best performance was obtained at a laser power of 2500 W, followed by 2800 and 2200 W.

## 1. Introduction

In recent years, wear- and corrosion-resistant materials have been used in various industrial areas. However, the slurry flow unit breaks easily due to erosive wear and erosion–corrosion, thus a material that has comprehensive properties needs to be used. Boride-based cermet composite coatings consisting of a transition metal base matrix with dispersed hard phases, such as Mo_2_FeB_2_, WCoB, MoCoB, and Mo_2_NiB_2_, possess high hardness, high melting points, good wear and corrosion resistance, and good optical and thermal properties [1,2,3,4,5,6]. These cermet coatings have been applied to injection molding machine parts, cutters for heat sealers, bearings for sea water pumps, offshore engineering parts, and slurry flow units in the coal-mining industry [7,8]. In particular, as emerging and promising materials with superior strength, hardness, wear, and corrosion resistance, M_3_B_2_-type boride-based cermet coatings, with Mo_2_FeB_2_, Mo_2_NiB_2_, and W_2_NiB_2_ as hard phases, are qualified candidates for applications that require wear and corrosion resistance [9,10]. 

Kenichi Takagi prepared the ternary boride using the sintering method, and systematically studied the effects of the Mo/B atomic ratio and additional elements on the mechanical properties and structure of boride cermets, and the mechanism of boride formation [11,12,13,14,15,16,17]. Cr and V addition resulted in improvement of the mechanical properties, such as TRS (transverse rupture strength) and hardness associated with a simultaneous phase transformation of complex boride from orthorhombic to tetragonal. The addition of Fe, Co, Ti, Mn, Zr, Nb, and W did not result in structural changes, but did degrade the mechanical properties [11]. Adding 3.5 wt.% Cr and 11.5 wt.% V, the TRS of boride increased when the Mo/B atomic ratio increased and then decreased, while the hardness and density increased as the ratio increased. 

The maximum TRS and hardness were 2.95 GPa and 90.5 R_A_ (Rockwell A hardness) [12]. The sintering mechanism of boride was analyzed by studying the microstructure of adding 10 wt.% Cr and 12.5 wt.% V, respectively, at different sintering temperatures, and the mechanical properties were discussed. The results showed that Mo_2_NiB_2_ was formed in the compact by a reaction of 2MoB + Ni = Mo_2_NiB_2_, and the addition of V yielded better mechanical properties [13]. The addition of Mn had an effect on the mechanical properties and microstructure of Mo_2_NiB_2_-based cermets in the powder composition of Mo, Ni, B, V, and x wt.% Mn. The hardness increased with Mn addition, while the TRS increased then decreased; the maximum TRS was 3.5 GPa at 2.5 wt.% Mn [14]. Adding 12.5 wt.% V and 2.5 wt.% Mn, the TRS of boride increased with the increasing Mo/B atomic ratio and then decreased, while the hardness increased as the ratio increased. 

The maximum TRS and hardness were 3.25 GPa and 90.8 R_A_ [15]. Adding V can greatly improve the mechanical properties of cermets. Corrosion tests in the molten fluorocarbon resin revealed that the Mo_2_NiB_2_ boride cermets had far better corrosion resistance than high-speed steel and SUS 304 [16]. The effects of Cr on the properties of Mo_2_NiB_2_ ternary boride were studied, while the Mo_2_NiB_2_ molar formula was assumed as (Mo_2−x_Ni_1−x_Cr_2x_)B_2_ for Cr-substituted Mo and Ni. Cr was doped into Mo_2_NiB_2_ ternary borides. In particular, 10 and 15 wt.% Cr exhibited high hardness and high elastic moduli, which makes them a suitable alternative material for wear-resistant hard materials, such as WC [17]. Yuan et al. [18] prepared Mo_2_NiB_2_ cermets and found that the maximum bending strength and hardness of Mo_2_NiB_2_ cermets reached 1.85 ± 0.04 GPa and 85.7 ± 0.1 R_A_, respectively. 

In Lei Zhang’s study, Mo_2_NiB_2_-Ni cremets with different Ni contents and ball milling times were fabricated by reaction boronizing sintering [19,20]. The cermets when molar ratio of Ni/B is 1.1 had the best mechanical properties and the lowest wear rates. The cermets with a milling time of 11 h exhibited a maximum hardness and bending strength of 87.6 HRA and 1367.3 MPa, respectively. Further, the high-temperature compressive strength and tribological behaviors of the cermets were investigated [21]. Sevinch [22] prepared Mo_2_NiB_2_ by self-propagating high-temperature synthesis using a mixture of MoO_3_, NiO, B_2_O_3_, and Al powders. 

Most research has focused on the sintering method. Wu et al. [23,24] found that continuous, dense, and adherent Mo_2_NiB_2_ cermet coatings were obtained by a laser surface cladding technique, which was chosen as laser cladding possesses some advantages over other methods, such as its convenience, high efficiency, high energy, cost effectiveness, and the fact that it is environmentally friendly [25]. However, the influence of laser parameters has not been deeply discussed in laser cladding synthesis. The laser power, as one of the most important variants, should be primarily investigated, as it would increase the scope of applications of Mo_2_NiB_2_ coatings. 

Herein, several various laser powers were studied to reveal the influences of power on the microstructure and properties of M_3_B_2_ boride-based cermet coatings on Q235 steels. Then, the microstructures of the coatings were analyzed by X-ray diffraction (XRD), scanning electron microscopy (SEM), and energy-dispersive X-ray spectroscopy (EDS). The hardness, corrosion resistance, and wear resistance of the coatings were evaluated. How the laser power affected the microstructure and properties of the coatings was revealed.

## 2. Materials and Methods 

### 2.1. Laser Cladding Synthesis

Commercially available Mo (Tianjiu, Changsha, China, 99.9% pure, particle size < 48 μm), B (Tianjiu, Changsha, China, 99% pure, particle size < 48 μm), Cr (Tianjiu, Changsha, China, 99% pure, particle size < 48 μm), and Ni60 (Kenna Metal, Pittsburgh, PA, USA, particle size < 45 μm) powders were used as raw materials. The composition of mixed powders is listed in Table 1. The Q235 steel (C 0.14%–0.22%, Mn 0.3%–0.65%, Si ≤ 0.3%, S ≤ 0.05%, and P ≤ 0.045%) was used as substrate with a size of 100 mm × 80 mm × 10 mm. 

**Figure 1:**
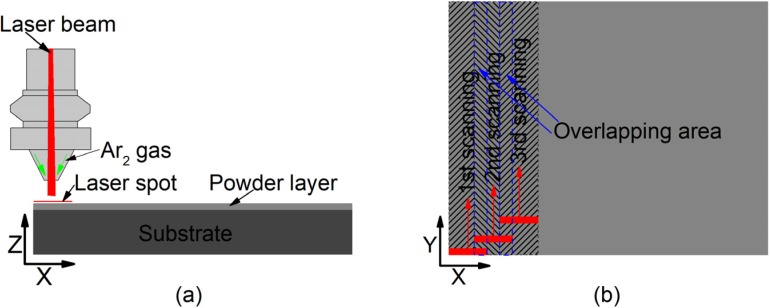
Laser cladding synthesis: (**a**) X–Z direction view; (**b**) X–Y direction view.

The raw powders were ball milled for 12 h in a planetary ball mill machine. To remove rust, dust, and oils, the substrates of Q235 steel were sandblasted for 10 min, and then cleaned with 99.8% alcohol in an ultrasonic cleaner for 15 min. As one of the common methods, the pre-placed powder method has been utilized here to investigate the effect of laser power on the properties of coatings. The mixed powders were placed on the surface of the substrate using polyvinyl butyral (PVB) binder. The thickness of the placed layer was about 1 mm. Then, the substrate layer was dried naturally in a drying cabinet. A Rofin DC030 laser (Rofin, Hamburg, Germany) was used as the laser source. The wavelength was 10.6 μm with a rectangular spot of 6 mm × 2 mm. The substrate with the layer was scanned by a laser after drying, as shown in Figure 1. During the laser cladding synthesis process, argon was used as a protective gas to prevent oxidation. As clearly seen in Figure 1a, the argon is blown out of the nozzle shielding oxygen entering the molten pool. The laser scanning sequences are shown in Figure 1b, and the laser spot was overlapped.

Here, the laser powers were 2200, 2500, and 2800 W, respectively, which was decided by good surface conditions in the forgone investigations, while the scanning speed was 1 mm/s, and overlap rate was 33%. The samples of 2200, 2500, and 2800 W were marked as No.1, No.2, and No.3, respectively.

### 2.2. Microstructure Analysis

The phase compositions of the cermet coatings were analyzed by X-ray diffraction (XRD, Rigaku Ultima IV, Tokyo, Japan) with Cu-Kα radiation (λ = 1.54 A) operated at 40 kV and 30 mA. The detected diffraction angle (2θ) was scanned from 20° to 100° and the scanning speed was 5°/min. The results were obtained using the XRD analysis software X’Pert HighScore Plus (v1.1, Malvern Panalytical, Etten Leur, Netherlands). The microstructure and composition distribution of the cermet coatings were characterized by scanning electron microscopy (SEM, Hitachi TM3030, Tokyo, Japan) equipped with an energy-dispersive X-ray spectroscopy (EDS, Oxford Swift 3000, Oxford, UK) machine.

### 2.3. Properties Analysis

The microhardness of the cross-sections of the cermet coatings was measured with a Vickers hardness tester (HXD-1000TMC/LCD, Shanghai TaiMing, Shanghai, China) under a load of 0.98 N and a dwell time of 15 s at room temperature. The corrosion resistance of the cermet coatings was evaluated by an electrochemical workstation (Autolab PGSTAT302N, Herisau, Switzerland) in 3.5 wt.% NaCl solution at room temperature. A standard three-electrode system was used. The reference electrode was a silver chloride electrode (Ag/AgCl), the counter electrode was platinum, and the working electrode was coated with specimens with a surface area of 1 cm^2^. Before each corrosion experiment, the cermet coatings were immersed in 3.5 wt.% NaCl solution for 1.5 h. The open circuit potential (OCP) was measured for 1 h. 

The electrochemical impedance spectra (EIS) was measured at the OCP with a potential amplitude of 5 mV and a frequency of 0.01 to 100,000 Hz. Potentiodynamic polarization experiments were scanned at 1 mV/s in the range of OCP ± 0.8 V, and the corrosion potential (E_corr_) and corrosion current (i_corr_) were obtained by the Tafel extrapolation method [26]. The corrosion results were analyzed by the software Nova 2.1.4 (version 2.1.4, Metrohm Autolab, Utrecht, The Netherlands). The wear resistance of the cermet coatings was measured by tribometer (UMT TriboLab, Bruker, Campbell, CA, USA). The cermet coatings reciprocated sliding against a Si3N4 ball with ϕ8 mm at room temperature under dry sliding conditions. The experiments were continuously applied with a load of 45 N lasting 1 h and a sliding frequency of 5 Hz for a sliding distance of 6 mm. The surface features of the cermet coatings were measured using a 3D optical microscope (ContourGT-1, Bruker, Campbell, CA, USA). After wear testing, the worn morphologies were observed by SEM.

## 3. Results

### 3.1. Microstructure and Composition of Cermet Coatings

**Figure 2:**
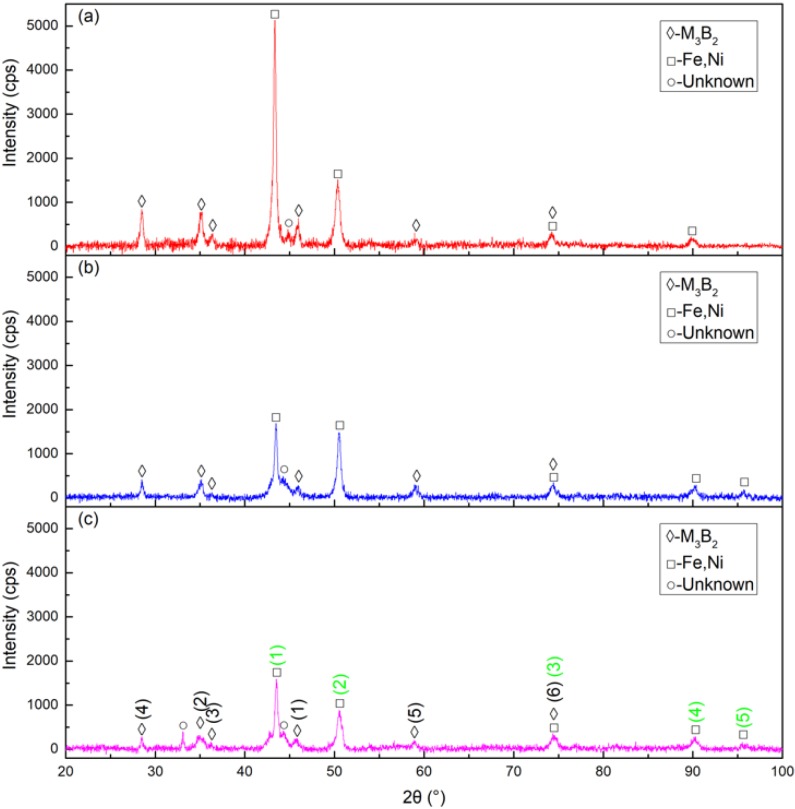
X-ray diffraction (XRD) patterns of the cermet coatings at various laser powers: (**a**) laser power of 2200 W; (**b**) laser power of 2500 W; (**c**) laser power of 2800 W.

The XRD patterns of cermet coatings were recorded. The laser power was increased to analyze the change in crystal structures. Figure 2. shows the XRD patterns of the coatings. The (M_2_M)_3_B_2_-type boride phase, {Fe, Ni} phase and unknown phase were detected in Sample 1, Sample 2, and Sample 3. The M in the (M_2_M)_3_B_2_ phase represents Mo, Ni, Cr, and Fe. They also represent the sorting of the intensity of the M_3_B_2_ and {Fe, Ni} phases, respectively, in a standard card with the black numbers and green numbers. The intensity of M_3_B_2_ in Sample 1 was much stronger than in Sample 2, while the intensity of Sample 2 was a little stronger than that of Sample 3. The intensity of {Fe, Ni} in Sample 1 was much stronger than in Sample 2 and 3 at position (1). The intensities of Sample 1 and Sample 2 were approximately the same at position (2) but much stronger than that of Sample 3. Position (1) was the strongest peak and position (2) was the second strongest peak in the standard card. The peak intensities of all samples were similar at the other three positions. The M_3_B_2_ phase intensity decreased as the laser power increased. The intensity of the {Fe, Ni} phase also decreased, but not sharply, as the laser power increased.

**Figure 3:**
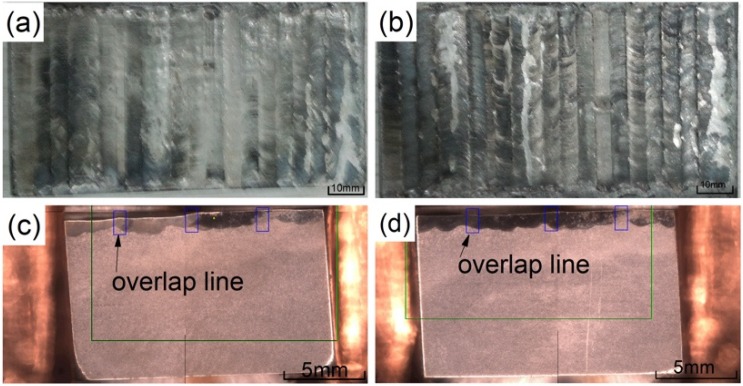
The macro morphology of the coatings: (**a**) 2500 W; (**b**) 2800 W; (**c**) cross section at 2500 W; (**d**) cross section at 2800 W.

Figure 3a,b show a macro view of the coating after cladding. It can be seen that the overall coating after lapping is relatively flat and there are no visible macro cracks. Figure 3c,d are SEM sample images after aqua regia corrosion is taken from the coating. The light gray is the substrate and the dark gray is the coating. The coating and the substrate are well combined, and the coating can be seen with detailed overlapping patterns.

**Figure 4:**
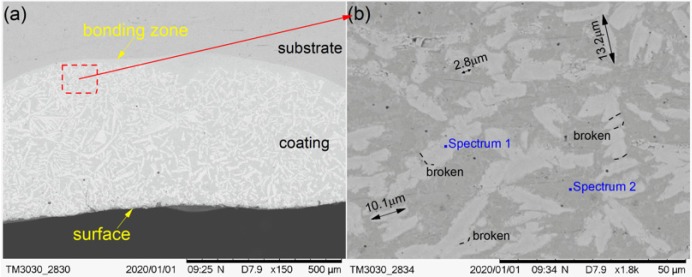
Scanning electron microscopy (SEM) morphology of the cermet coatings at 2200 W: (**a**) low magnification; (**b**) high magnification.

**Figure 5:**
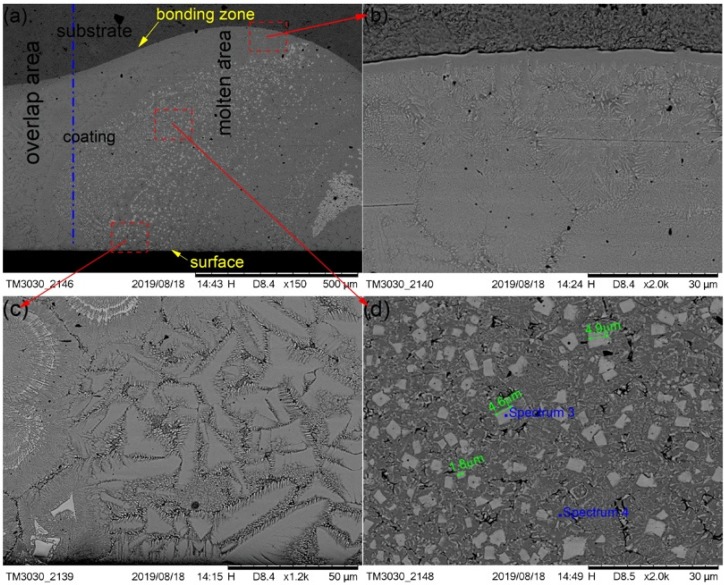
SEM morphology of the cermet coatings at 2500 W: (**a**) low magnification; (**b**) bonding zone at high magnification; (**c**) surface area at high magnification; (**d**) middle area at high magnification.

**Figure 6:**
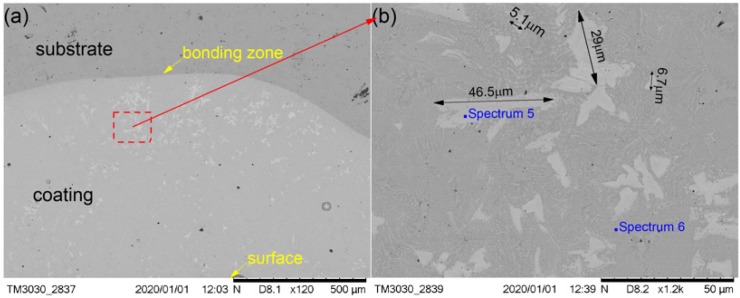
SEM morphology of the cermet coatings at 2800 W: (**a**) low magnification; (**b**) high magnification.

The cross-section morphological images of cermet coatings were recorded with the increase of laser power, as shown in Figure 4, Figure 5 and Figure 6. There is an interface between the substrate and the coating in the morphological images. Distributed in cermet coating Sample 1, Sample 2, and Sample 3, there are white and gray phases, which are the M_3_B_2_ hardness phases and {Fe, Ni} binder phases, respectively. The curve radian of the interface at the molten pool area is evidently larger than that of the overlap area shown in Figure 4a. The white phase is long and uniformly distributed in the coating, as shown in Figure 4, while the laser power was 2200 W. Compared with the gray phase in the coating, the white phase is more abundant. The smaller grain size is about 2.8 μm, the larger grain size is about 10 μm, and the much longer grains will break, as shown in Figure 4b.

Figure 5 shows the SEM images that depict the cross-sectional morphology of the cermet coatings at the laser power of 2500 W, and shows enlarged views of the bonding zone (Figure 5b), the surface area (Figure 5c), and the middle area (Figure 5d), respectively. The curve radian of the interface at the laser power of 2500 W is larger than that of 2200 W. The curve radian of the bonding zone at the molten pool area is clearly larger than that of the overlap area. Figure 5a shows that the white phase is evenly distributed in the molten pool area, and less distributed in the overlap area and the area near the interface. Compared with the grain at 2200 W, the grain size at 2500 W was significantly smaller and more regular. However, the distribution of grains in the entire cross-section of the coating was more uniform at 2200 W. 

It can be seen from Figure 4a and Figure 5a that the white phase was present in a lower quantity at 2500 W than that at 2200 W. The morphology of the gray phase is different in the bonding zone and the surface area. In the surface area, the gray phase is constituted by crystals that are shaped as triangles, squares, bars, and other shapes, and the edges have burrs similar to feathers. In the interface area, most of the gray phases contain crystals that are square-shaped, and some of them contain thin, long white phases. It can be seen from Figure 5d that the shape of crystals in the white phase in the middle area is basically square, the small grain size is approximately 1.8 μm, and the largest grain size is approximately 4.9 μm. 

Figure 6 shows the SEM images depicting the cross-sectional morphologies of the cermet coating at the laser power of 2800 W and shows enlarged views of the middle area (Figure 6b). As in Figure 4a and Figure 5a, the curve radian of the interface at the molten pool area is larger than that of the overlap area in Figure 6a. The white phase was present in a lower quantity, and it was unevenly scattered in the coating at 2800 W. There are more white phases distributed at the bottom of molten pool than other areas. The morphology of the white phase is irregular, as shown in Figure 6b. The smaller grain size was approximately 5.1 μm, the larger grain size was approximately 29 μm, and the long grain size could reach up to 50 μm.

**Figure 7:**
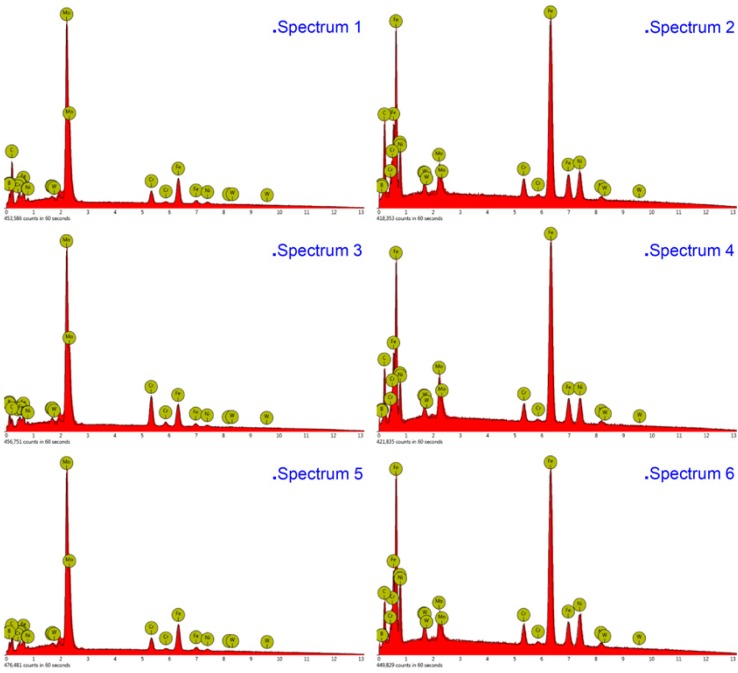
Energy-dispersive X-ray spectroscopy (EDS) analysis of the white and gray phases in the cermet coatings at various laser powers.

The compositions of the white and gray phases—marked as spectrums 1 to 5 in Figure 4, Figure 5 and Figure 6—were evaluated, as shown in Figure 7, with the increase of laser power. In order to elucidate the effects of laser power on the elements of the white phases, the EDS spectrums 1, 3, and 5 were employed in three kinds of cermet coatings, respectively. Meanwhile, spectrums 2, 4, and 6 were also carried out to depict the variation of gray phases in the cermet coatings. Evidently, the element contents of the white phase and the gray phase are completely different, as shown in Figure 7. The contents of Mo are much higher in the white phases than in the gray phases, whereas the contents of Fe are greatly increased in the gray phase. Thus, the gray phases can be considered as the binder of {Fe, Ni} with few Mo, and the white phases are M_3_B_2_ with B aggregation, in which M represents Mo, Ni, Cr, and Fe. The high concentration of Fe originates from the dilution of substrate steels, which is a common phenomenon in laser cladding [27]. The detailed element contents are given in Table 2.

### 3.2. Hardness of M_3_B_2_-Based Boride Cermet Coatings

**Figure 8:**
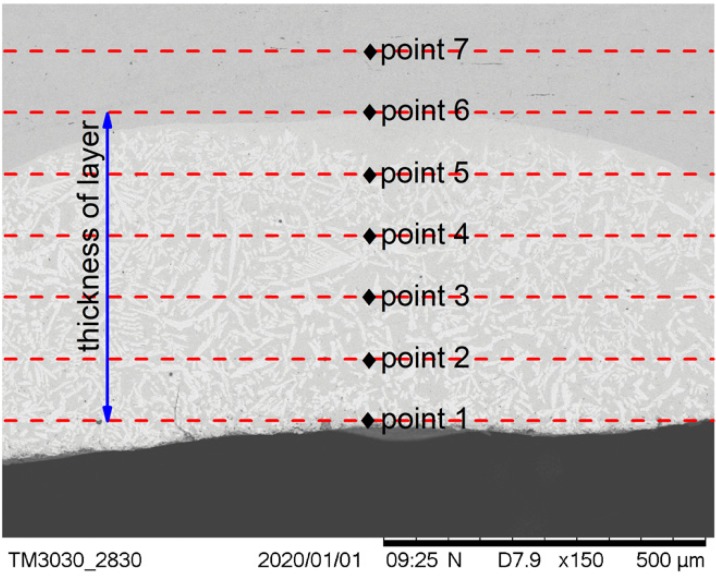
Positions of the hardness measurements.

The positions at which the micro Vickers hardness tests of the cermet coatings were carried out are shown in Figure 8. Seven points were tested on the cross-section, and the first point was taken as close as possible to the coating surface. The distance between the test points was one fifth of the thickness of the coating. The distance between the test points was different in the three samples as the coating thicknesses of the samples were different. 

**Figure 9:**
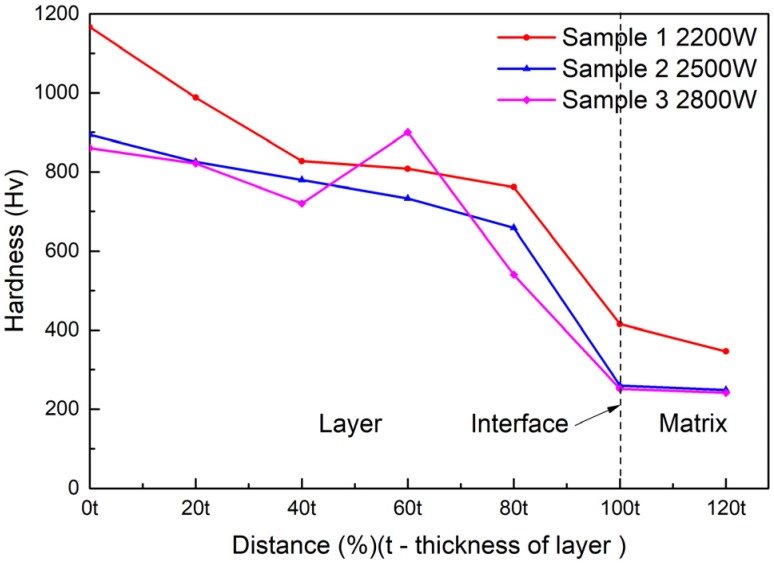
Vickers hardness of the cermet coatings at various laser powers.

The results of the hardness test are shown in Figure 9. This indicated that the micro-hardness decreased along the surface of the substrate at different laser powers. The hardness of the coating decreased with increasing laser power. The hardness at 2500 W was slightly greater than the hardness at 2800 W, but significantly less than the hardness at 2200 W. The hardness suddenly changed at a certain position in the middle area of the coating when the power was 2800 W; it was not only higher than the hardness of the two positions before and after it, but also higher than the hardness of the other two samples at the same position. The maximum hardness value in all test points was 1166.3 HV, when the power was 2200 W at the surface position. 

### 3.3. Corrosion Tests of Cermet Coatings

The substrate and all cermet coatings were immersed in 3.5 wt.% NaCl solution for 1.5 h before the OCP test. The results of the OCP were listed in Table 3. Compared to the coatings and substrate, the OCP shifted positively. The difference of the OCP between 2200 and 2800 W was not great, but the OCP reached −0.3161 V at 2500 W with a significant increase.

**Figure 10:**
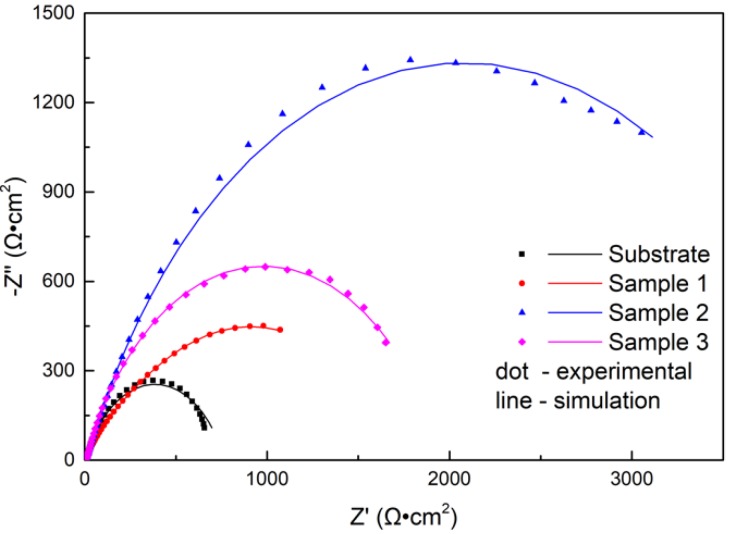
Nyquist diagrams for the substrate and cermet coatings at various laser powers.

As is known, the EIS technique has been extensively used to characterize surface coatings in a semiquantitative way [28]. Nyquist plots recorded with samples immersed in NaCl solution are shown in Figure 10. All impedance spectra of the samples displayed depressed semicircles. The diameter of the impedance spectra reflected the rate of the electrochemical reaction [29]. As shown in Figure 10, the diameter of the impedance spectra arc of coatings increased compared to the substrate. The laser power had a great effect on the impedance spectra of the coating, and the diameter of the impedance spectra arc increased first and then decreased with increasing power. When the power was 2500 W, the corrosion rate was minimum. 

**Figure 11:**
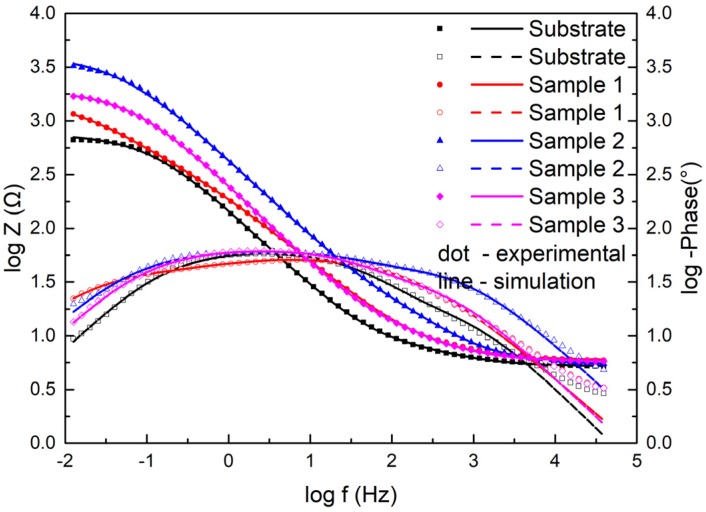
Bode diagrams for the substrate and cermet coatings at various laser powers.

Moreover, Figure 11 displays the electrochemical data of the samples as Bode plots. They exhibited the same features as the Nyquist plots. The Z value was the smallest for the substrate and the largest in Sample 2 among the three coating samples at the lowest frequency, which indicated that the coating had better corrosion resistance than the substrate and had the best corrosion resistance at 2500 W.

**Figure 12:**
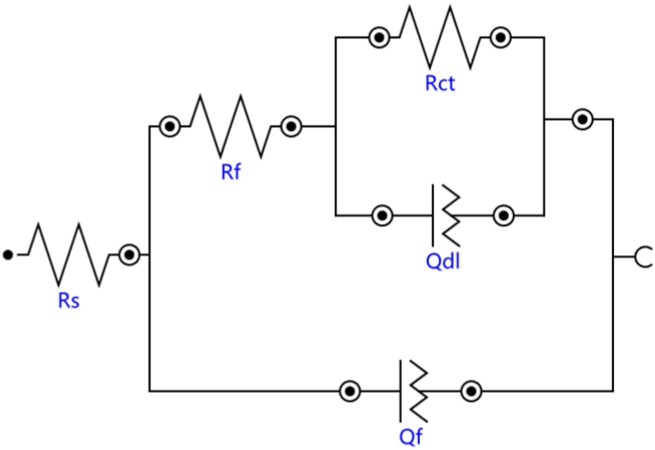
Equivalent circuit model of the substrate and cermet coatings.

The Bode diagrams with a wide phase angle in Figure 11 indicated that there are at least two time constants [30]. The equivalent circuit of the two time constants in Figure 12 was used to fit the EIS data using Nova software. Here, R_s_ is the solution resistance from the reference electrode to the working electrode, R_f_ is the surface coating resistance, and R_ct_ is the constant phase elements of the charge transfer resistance. The impedance of the regular phase-angle element Q is related to the angular frequency (ω) by the following relation: Z_CPE_ = Y_0_^−1^(jω)^−n^, where Y_0_ is a proportionality factor and n is the deviation parameter, which reflects the roughness of the surface; Q_f_ is the constant phase angle element (CPE) between the solution and the surface, and Q_dl_ is the CPE of the interface double-layer between the solution and the matrix. The fitting lines are also plotted in Figure 10 and Figure 11, and the fitting results are in good agreement with the experimental results, indicating that the equivalent circuit is feasible.

The fitting results of the EIS data of all samples immersed in 3.5 wt.% NaCl solution with different processing parameters are listed in Table 4. The solution resistance R_s_ did not change significantly, indicating that the environmental conductivity was relatively stable. Compared with the substrate, the R_f_ of coatings increased, and the R_f_ at 2200 W was much larger than at 2500 W and 2800 W, which indicated that laser power had an effect on the surface coating resistance. The polarization resistance R_p_ (R_p_ = R_f_ + R_ct_) is used to evaluate the corrosion resistance of materials [31]: the greater the R_p_ value, the better the corrosion resistance. The R_p_ of Sample 2 was 4115.6 Ωcm^2^, more than twice that of Sample 1 and Sample 3, and the R_p_ of Sample 3 was slightly larger than that of Sample 1. The results are consistent with the previous analysis of the Nyquist and Bode plots.

**Figure 13:**
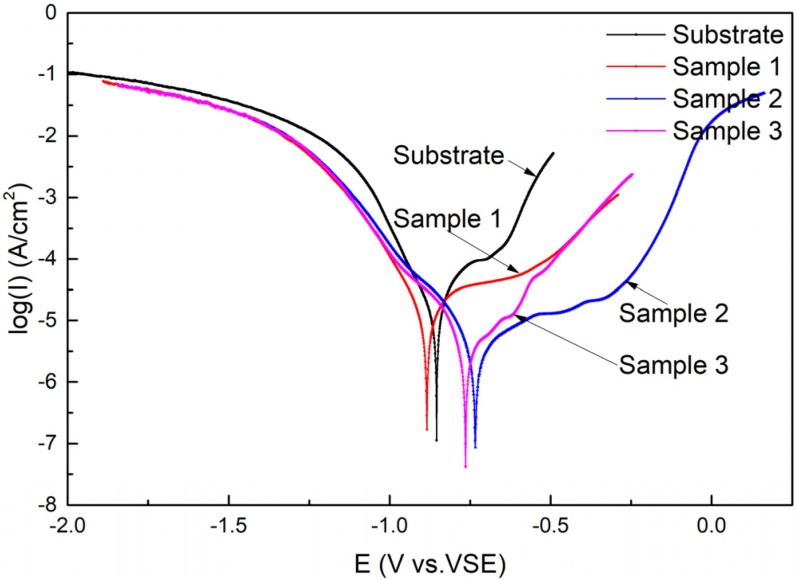
Polarization curves of the substrate and cermet coatings.

The polarization curves of the substrate and cermet coatings immersed in 3.5 wt.% NaCl solution are presented in Figure 13. It is evident from Figure 13 that both the substrate and the coatings have undergone the transition process from the activated state to the passivated state and ultimately, to overpassivation; however, the duration of each process varied. The self-corrosion potentials of Samples 2 and 3 shifted positively compared to the substrate, but the self-corrosion potentials of Sample 1 shifted negatively, while the OCPs of coatings were all positively shifted. The passivation state of the substrate and Sample 3 was short, quickly changing to the overpassivation state, but Sample 3 experienced a brief secondary passivation state. Samples 1 and 2 maintained a long passivation state, and Sample 2 entered a secondary passivation state after overpassivation. The polarization curves show that the coating at 2500 W had very good corrosion resistance.

According to the relationship between the current density i and the polarization potential E of the Butter–Volmer equation (Equation (1) and (2)), the polarization resistance R_p_ can be calculated by Equation (3) [32].

i = i_corr_[exp(2.303ΔE)/b_a_ − exp(−2.303ΔE)/b_c_], (1)

ΔE = E − Ecorr(2)

R_p_ = b_a_b_c_/(2.303(b_a_ + b_c_)i_corr_). (3)

The corrosion parameters related to the polarization curves are calculated and listed in Table 5. i_corr_ is the corrosion current density, E_corr_ is the self-corrosion potential, b_a_ is the anode Tafel constant, and b_c_ is the cathode Tafel constant. E_corr_,Obs in Table 5 is the observed value, and E_corr_,Calc is the calculated value. The i_corr_ decreased from 16.9 to 2.93 μA, which indicated that the coating on the substrate greatly improved the corrosion performance of the substrate. As the laser power increased, i_corr_ decreased and then increased, and the polarization resistance showed the opposite trend. The results of the analysis are consistent with the previous results.

### 3.4. Wear Tests of Cermet Coatings

**Figure 14:**
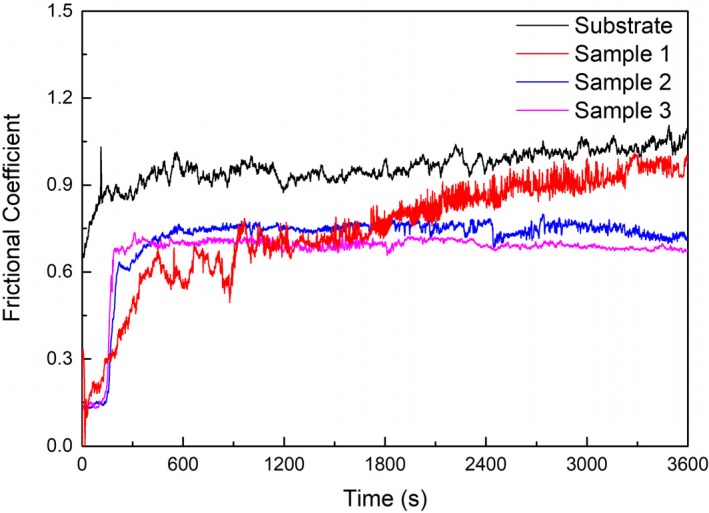
Friction coefficient of the substrate and cermet coatings.

The frictional coefficient curves of the substrate and cermet coatings are shown in Figure 14. The frictional coefficients of the substrate and coatings rose rapidly at the start of the test, and then the substrate and Samples 2 and 3 entered the steady wear stage after a breaking-in process of approximately 2.5 min, whereas Sample 1 took approximately 7.5 min. After entering the steady state, the frictional coefficient of the substrate and Sample 1 displayed some fluctuations and showed a slight upward trend, while the fluctuations in the frictional coefficient of Samples 2 and 3 were small and showed a downward trend. The order of the frictional coefficients of the substrate and the coating tended to be consistent after sliding for 30 min. The frictional coefficient of the substrate was larger than that of the coatings, and this decreased as the power increased. The frictional coefficient of coatings was the smallest at 2800 W.

**Figure 15:**
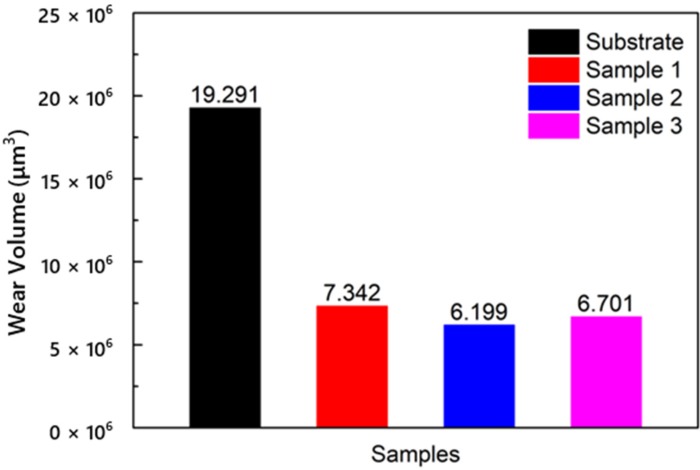
Volume losses of the substrate and cermet coatings at various laser powers.

The wear volumes of the substrate and cermet coatings are shown in Figure 15. The volume loss of the substrate was approximately 2.6 to 3.1 times that of the coatings. The influence of laser power on the volume loss of coatings was different from the frictional coefficient. The volume loss increased as the frictional coefficient increased. The frictional coefficient of Sample 2 was larger than that of Sample 3, but the volume loss of Sample 2 was smaller. The coating with the best wear resistance was obtained at a laser power of 2500 W.

**Figure 16:**
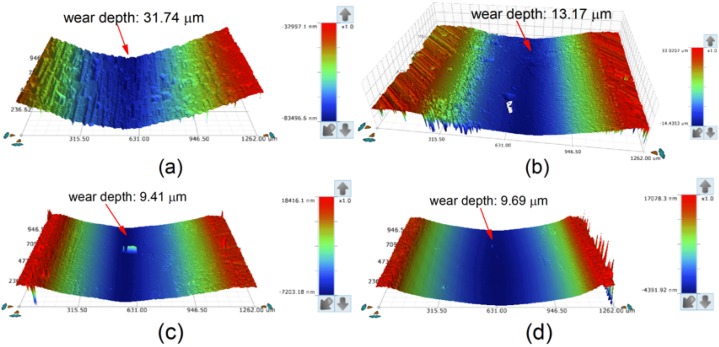
Three-dimensional topography of the wear tracks: (**a**) substrate; (**b**) laser power of 2200 W; (**c**) laser power of 2500 W; (**d**) laser power of 2800 W.

The three-dimensional morphologies of the wear tracks of the substrate and coatings are shown in Figure 16. The plow-furrows can be clearly identified in the worn area of the substrate, and some pits were found on the surface of Sample 1, while only fine scratches were found on the surfaces of Samples 2 and 3. As seen from Figure 16, the wear tracks of coatings had a lower wear depth than that of the substrate (31.74 μm). The wear depth of Sample 2 (9.41 μm) was similar to that of Sample 3 (9.69 μm), which was much smaller than that of Sample 1 (13.17 μm). This further confirmed that the coating had the best wear resistance at 2500 W.

**Figure 17:**
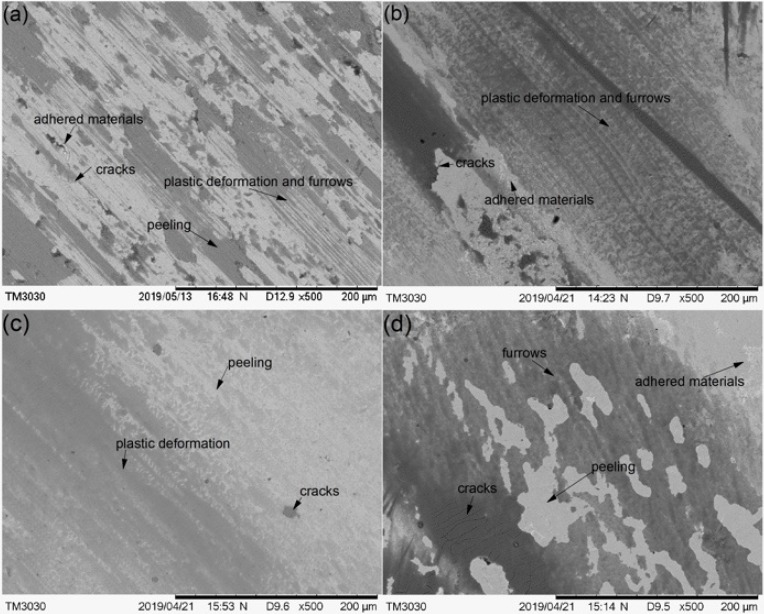
The SEM morphology of the worn substrate and cermet coatings: (**a**) substrate; (**b**) laser power of 2200 W; (**c**) laser power of 2500 W; (**d**) laser power of 2800 W.

The worn morphologies of the substrate and cermet coatings were observed by SEM, as shown in Figure 17. The worn surface of the substrate displayed large plastic deformation and furrows, and many adhered materials and microcracks parallel to the ploughing direction (Figure 17a). The worn surface of Sample 1 did not have obvious ploughing furrows, but had distorted plastic deformation, peeling and adhered materials, and micro-cracks appeared in the peeling layer (Figure 17b). The worn surface of Sample 2 was relatively flat, with small plastic deformation and peeling. Micro-cracks were observed in the peeling layer, and no obvious adhered materials were seen (Figure 17c). The worn surface of Sample 3 had shallow furrows and more peeling and adhered materials, and there were micro-cracks in the dark area (Figure 17d). The analysis is consistent with the previous results.

## 4. Discussion

The XRD analysis showed that there were at least two phases in the coating. By comparison with the standard card, the two phases were identified as M_3_B_2_ and {Fe, Ni}. The absolute intensity of M_3_B_2_ in Sample 1 was much stronger than in Sample 2, while the intensity of Sample 2 was slightly greater than that of Sample 3. The absolute intensity of {Fe, Ni} in Sample 1 was also much stronger than in Sample 2, but the intensity of Sample 2 was a little stronger than that of Sample 3. From the XRD diffraction peaks of the samples, their integrated intensity changes were the largest at 2200 W, the second largest at 2500 W, and the smallest at 2800 W. Therefore, according to the XRD phase calculation method, the change in the content of the M_3_B_2_ phase was the largest at 2200 W, followed by 2500 W, and the smallest change was observed at 2800 W. It can be seen from the SEM that the coatings consisted of a white phase and gray phase. The results of EDS analysis of the white and gray phases indicated that the white phase was M_3_B_2_ and the gray phase was {Fe, Ni}. Fe and Cr occupied the positions of the Ni atoms in the white phase M_3_B_2_ [21,24]. 

The M_3_B_2_ phase was long and uniformly distributed in the coating at 2200 W. Compared with the gray phase in the coating, the white phase was present in a greater quantity. The M_3_B_2_ phase was evenly distributed in the molten pool area, and less distributed in the overlap area and the area near the interface at 2500 W. The M_3_B_2_ phase was present in a smaller quantity, and it was unevenly scattered in the coating at 2800 W, while there was a greater amount of M_3_B_2_ phase distributed in the bottom of the molten pool than other areas. The smaller grain size was approximately 2.8 μm, and the larger grain size was approximately 10 μm at 2200 W. The small grain size was approximately 1.8 μm, and the largest grain size was approximately 4.9 μm at 2500 W. The smaller grain size was approximately 5.1 μm, the larger grain size was approximately 29 μm, and the long grain size could reach 46.5 μm at 2800 W. 

When the laser power was changed, the energy obtained per unit time, the depth, and disturbance of the molten pool, and the solidification time changed as a result. When the power was 2500 W, the molten pool became deeper, and Fe in the substrate floated to the top of the molten pool; however, as the disturbance became larger and the cooling time became longer, the grain size became smaller. When the power was 2800 W, a large amount of Fe floated to the top of the molten pool, which changed the atomic ratio of the elements in the molten pool, resulting in a reduction in the quantity of M_3_B_2_ phase that was formed. The complex spatial microstructures of the coatings including the complex-shaped M_3_B_2_ and {Fe, Ni} phases were caused by the non-equilibrium solidification behavior of the laser cladding coating in the quick-cooling temperature filed, which has been investigated comprehensively in other works [33,34].

The improvement of the hardness of the cermet coatings can be explained by particle dispersion strengthening [35]. The M_3_B_2_ boride particles in the composite coatings acted as a strengthening phase that was distributed in the solid solution phase. M_3_B_2_ is a hard and brittle phase that is difficult to deform. The crystal structure of M_3_B_2_ is different from the {Fe, Ni} phase; therefore, when the dislocation cut through the particles, the slip resistance increased, which resulted in an increase in the hardness of the coating. It was found from Figure 9 that the hardness of the coating decreased as the laser power increased. The main reason for this result was the different volume fraction of the reinforcing particles in the coating. The volume fraction can be clearly seen in Figure 4a, 5a and 6a. The results of the hardness test show that the hardness gradually decreased from the surface to the bonding zone at 2200 W. Figure 4a shows the microstructure of the coating at 2200 W, the grain size in the surface area was smaller than that in the middle area, and fewer particles were present in the bonding area. The grain refinement can improve the performance according to the Hall–Petch strengthening formula (Equation (4)) [36].

σ_s_ = σ_0_ + Kd^−1/2^, (4)

where σ_s_ is the yield strength of the materials, σ_0_ is the deformation resistance of the grain, K is the influence coefficient of the grain on deformation, and d is the average size of the grain. The hardness of the coating at 2500 W decreased slightly from the surface area to the middle area, and decreased significantly in the bonding zone. Figure 5a shows the microstructure of the coating at 2500 W, the volume fraction and size of the grains did not change greatly in the surface and middle area but occupied a small proportion in the bonding area. Figure 9 shows the hardness of the coating at 2800 W gradually decreased from the surface area to the bonding area, but suddenly increased in the middle area approaching the bonding area. The sudden increase in hardness caused by a large amount of M_3_B_2_ phase gathered at that area, which can be evidently seen in Figure 6a. Figure 6a shows the microstructure of the coating at 2800 W, the grain size and volume fraction of the second phase in the surface and middle area were small, but they were large at test position 4. The second phase distribution in the coating was different, due to the differences of melting depth, disturbance, and cooling time of the molten pool under different laser powers.

The EIS test results are shown in Figure 10 and Figure 11, which show that the corrosion performances of the coatings were much better than that of the substrate. The corrosion resistance of the coating was different at different laser powers; it was highest at 2500 W, followed by at 2800 W and lowest at 2200 W. The EIS plots were fitted by the Nova software, and the model R_s_(Q_f_(R_f_(Q_dl_R_ct_))) was applied to analyze the variations in corrosion. Both R_f_ and R_ct_ increased, indicating that the surface properties of the substrate were changed with the coating, and the corrosion performance was improved. The R_f_ coating at 2200 W was much larger than that of coatings 2 and 3, due to the difference of the boride ceramic phase volume fraction. The law of R_f_ at various powers was consistent with the law of the volume fraction of boride ceramic phase M_3_B_2_ in the coating. In the EIS diagram, the high-frequency part is related to the charge transfer, which is associated with the effect of an electric double-layer [37]. The R_ct_ of the coating at 2500 W was larger than the others, which means that the electric double-layer formed by the structure of the coating at 2500 W was more likely to hinder the electron transfer in 3.5 wt.% NaCl solution. 

The polarization curves show that both the substrate and the coatings underwent the transition process from the activated state to the passivated state and ultimately, to overpassivation; however, the duration of each process varied. The corrosion resistance was improved by the coatings compared to the bare substrate. The corrosion current density i_corr_, the Tafel constant for an anode b_a_, and the Tafel constant for a cathode b_c_, which reflect the corrosion mechanism and kinetics are considered as electrochemical corrosion parameters [38]. The absolute values of b_a_ are greater than those of b_c_, indicating that the anode is the control step of the whole electrochemical reaction. The corrosion mechanism of substrate and coatings in 3.5 wt.% NaCl solution can be deduced as follows.

Anodic reaction:

(5) $${M=M}^{n+}{+ne}^{-},$$

Cathodic reaction:

(6) $$\frac{n}{2}H_{2}O+\frac{n}{4}O_{2}{+ne}^{-}{=nOH}^{-}$$

where M is the metal elements in the coating (according to the XRD and SEM analyses, M is Fe and Ni), and n^+^ is the valence of elements. The formed passivation film can reduce the average speed of the anode dissolution process, which was characterized in Hamadou’s study [39]. The polarization curves show that the passivation film formed on the substrate and the coatings, but the electrode reaction of the substrate was faster, and the film was quickly dissolved. The electrode material and surface state had a significant effect on the reaction speed of the electrode. The microstructure of the coatings was different, which caused the differences in the coatings’ abilities to hinder electron transfer, and the coating at 2500 W was the strongest according to the EIS results. 

The volume fraction of {Fe, Ni} was large in the surface area of Sample 3, and the short maintenance time of the passivation state was similar to that of the substrate. However, the M_3_B_2_ phase in the coating caused the potential to shift positively, and the corrosion resistance was improved. The volume fraction of M_3_B_2_ was larger in the surface area of Coatings 1 and 2, the passivation state was maintained in this microstructure. However, the dense M_3_B_2_ phase was distributed in the {Fe, Ni} phase, which made it easier to form galvanic cells between them, which accelerated the corrosion process.

The tribological properties are not inherent properties of the materials and depend on the mechanical properties and some other factors, such as the tribo-phase, roughness, and toughness [40]. The substrate and coatings were ground under the same process conditions to ensure consistent roughness. The decrease of the frictional coefficient and wear rate of the coatings compared to the substrate due to the hardness improved according to Archard’s principle [41]. 

The substrate underwent abrasive wear and adhesion wear demonstrated by the furrows and adhered materials on the worn track (Figure 17a). The M_3_B_2_ phase in the coating resisted deformation, so that there were no obvious furrows in the wear track, and the abrasive wear was reduced. The hardness of Sample 1 was greater than that of Samples 2 and 3; however, the frictional coefficient and volume loss were greater, because the volume fraction of the M_3_B_2_ phase in Sample 1 was large, and a small amount of the bonding phase was insufficient to protect the hard particles during the friction process. Then, the particles were peeled off (Figure 17b). 

The frictional coefficient of the coating at 2500 W was greater than at 2800 W in Figure 14, but the volume loss was smaller in Figure 15. Sample 3 displayed a large area of peeling during the friction process, but only a small amount of the peeling layer remained in the wear track, so the frictional coefficient was not large; however, the volume loss was large (Figure 17d). Fine, square-shaped M_3_B_2_ particles were uniformly distributed in Sample 2, which improved the hardness of the coating, and a sufficient bonding phase protected the M_3_B_2_ particles, thus, there were no obvious deformations, furrows, or adhesive materials in the wear track, only fewer peeling phenomena (Figure 17c).

## 5. Conclusions

In this study, M_3_B_2_ boride-based cermet coatings were developed on Q235 steel by laser cladding synthesis using different laser powers. The laser power had significant effects on the microstructure, and the properties of the coatings were studied. The coating consisted of a white, hard M_3_B_2_ phase and a gray bonding phase {Fe, Ni}. The laser power increased and the grains in the coating were refined; however, the uniformity of the distribution decreased due to the increase of the molten pool disturbance. When the power was further increased, the dilution rate increased, and a large amount of Fe entered the coating from the steel. The thickness of the coating increased, but the amount of hard M_3_B_2_ phase in the coating decreased.

The hardness of the coatings demonstrated a linear relationship with the power, that is, the hardness decreased with the increase of power, because the increase in power caused the precipitation of Fe in the steel and then the reduction of the proportion of the hard phase in the coating. The maximum hardness of the coating was 1166.3 HV, which was obtained at 2200 W.

From the EIS and polarization curve analysis, it was shown that the corrosion performance of the coating was much better than that of the substrate. The corrosion current of the coatings was reduced by an order of magnitude compared to the substrate, and the polarization resistance was increased by an order of magnitude. The corrosion performance of the coatings first increased and then decreased with increasing power. The coating with the best corrosion performance was obtained at a power of 2500 W, at which time the corrosion current was 2.93 μA, and the polarization resistance was 11949 Ω. 

The frictional coefficient and wear loss of the coatings were lower than those of the substrate. The wear performance was also better. Under the same conditions, the volume loss of the substrate was 19.29 μm^3^, and the maximum volume loss of the coating was 7.34 μm^3^, which greatly improved the wear resistance. The frictional coefficient decreased with increasing power, while the volume loss was reduced and then increased. The volume loss showed that the coating had the best wear resistance when the power was 2500 W. 

The prepared coating greatly improved the overall performance of the substrate. The key process parameter—laser power—should not be too low or too high, but the performance at high power was better than that at low power. At 2500 W, the coating demonstrated not only the best corrosion resistance but also the best wear resistance. These cermet coatings can be used in the manufacture and repair of mining machinery parts, seawater pump parts, offshore engineering parts, and slurry flow units.

## Figures and Tables

**Table 1:** Composition of the powder.

Element	Mo	Ni	B	Cr	Fe	Si	W	C
wt.%	57.82	17.68	7.58	10	4.56	1.21	0.91	0.24

**Table 2:** Element contents (at%) at different locations from EDS.

Laser Power (W)	Location	Mo	B	Ni	Fe	Cr	Si	W
2200	Spectrum1	40.81	28.43	2.32	20.42	6.72	0.76	0.54
	Spectrum2	4.29	0	17.23	71.11	4.38	2.33	0.66
2500	Spectrum3	40.76	28.56	2.11	20.96	6.72	0.62	0.27
	Spectrum4	3.95	0	19.34	68.08	4.72	3.25	0.66
2800	Spectrum5	36.21	30.26	1.48	15.83	14.19	1.2	0.84
	Spectrum6	5.64	0.74	15.71	71.08	4.23	2.22	0.39

**Table 3:** The open circuit potential (OCP) of the substrate and cermet coatings immersed in NaCl solution.

	Substrate	Sample 1	Sample 2	Sample 3
OCP (V)	−0.6438	−0.54443	−0.3161	−0.52393

**Table 4:** Fitted values of the equivalent circuit of the electrochemical impedance spectra (EIS) diagram.

Sample No	R_s_ (Ωcm^2^)	R_f_ (Ωcm^2^)	Q_f_,Y_0_ (mΩ^−1^cm^−2^s^n^)	R_ct_ (Ωcm^2^)	Q_dl_,Y_0_ (m^−1^cm^−2^s^n^)
Substrate	5.21	5.39	0.675	751	0.952
No.1	5.9	639	1.342	1072	2.165
No.2	5.35	48.6	0.397	4067	0.197
No.3	5.78	20.2	0.659	1928	0.323

**Table 5:** The electrochemical parameters obtained from the polarization curves of the substrate and coatings.

Sample No	E_corr_,Obs (V)	E_corr_,Calc (V)	i_corr_ (A)	|b_a_| (V/dec)	|b_c_| (V/dec)	Polarization Resistance (Ω)
Substrate	−0.85436	−0.87408	1.6907 × 10^−5^	0.17169	0.09951	1618.2
No.1	−0.88434	−0.89038	1.1189 × 10^−5^	0.22088	0.10775	2811
No.2	−0.73426	−0.72866	2.9279 × 10^−6^	0.24416	0.12023	11949
No.3	−0.76463	−0.74899	3.2315 × 10^−6^	0.2036	0.12814	10569
